# Short-Packet Communications in Multi-Antenna Cooperative NOMA Networks with Hardware Impairments

**DOI:** 10.3390/s25175444

**Published:** 2025-09-02

**Authors:** Xingang Zhang, Dechuan Chen, Jianwei Hu, Xiaolin Sun, Baoping Wang, Dongyan Zhang

**Affiliations:** 1 School of Physics and Electronic Engineering, Nanyang Normal University, Nanyang 473061, China; 2 Collaborative Innovation Center of Intelligent Explosion-Proof Equipment, Henan Province, Nanyang 473061, China; 3 Faculty of Education, Henan Normal University, Xinxiang 453007, China; 4 National Key Laboratory for Complex Systems Simulation, Beijing 100101, China

**Keywords:** effective throughput, multi-antenna, NOMA, short-packet communications

## Abstract

This work examines the performance of a multi-antenna cooperative non-orthogonal multiple access (NOMA) network that employs short-packet communications and operates under the effect of hardware impairments. Specifically, a multi-antenna source transmits superposition-coded NOMA signals to a near user and a far user. Acting as a decode-and-forward (DF) relay, the near user adopts successive interference cancellation (SIC) to decode and subsequently forward the message intended for the far user. In addition, the transmission strategy at the source is the maximum ratio transmission (MRT) and the reception strategy at the far user is selection combining (SC). For Nakagami-*m* fading channels, closed-form expressions for the average block error rate (BLER) and effective throughput are derived. Then, the effective throughput is maximized through the optimization of the blocklength, accounting for constraints on transmission latency and reliability. The results obtained from simulations confirm the analytical findings and demonstrate that the proposed scheme, with a two-antenna source configuration, achieves a superior effective throughput, reaching up to 240% at a transmit signal-to-noise ratio (SNR) of 33 dB, compared to the existing NOMA scheme in the literature.

## 1. Introduction

The massive connectivity requirements of Internet of Things (IoT) networks present significant challenges for multiple access technique design in wireless communication networks [1,2,3]. As a promising solution, non-orthogonal multiple access (NOMA) has emerged as a critical multiple access technique for future wireless networks, offering substantial potential to address the massive connectivity demands in IoT applications [4,5,6]. In power-domain NOMA networks, a base station simultaneously serves multiple users on the same time-frequency resource through differentiated power allocation. To mitigate co-channel interference, receivers employ successive interference cancellation (SIC) to decode their intended messages. Compared with orthogonal multiple access (OMA), NOMA demonstrates superior performance in several key aspects, including enhanced spectral efficiency, reduced transmission latency, and improved user connectivity [7,8,9].

### 1.1. Background

The incorporation of relay nodes into NOMA networks can further enhance transmission reliability and radio coverage [10]. Specifically, in NOMA networks, a user with better channel conditions has to decode signals intended for a user with poor channel conditions before decoding its own signal. Consequently, leveraging the user with better channel conditions as a relay node becomes an effective strategy to boost the signal reception performance of the user with poor channel conditions. In [11], the authors examined full-duplex user relaying in a cooperative NOMA network. Additionally, to incentivize users with better channel conditions to participate in cooperative relaying, ref. [12] incorporated simultaneous wireless information and power transfer into NOMA networks. Then, Terahertz (THz) communications were combined with NOMA in [13,14] to substantially improve spectral efficiency and support massive connectivity, utilizing ultra-broad bandwidth and advanced multi-user multiplexing techniques. Specifically, ref. [13] proposed a channel-aware mechanism for simultaneous wireless information and power transfer (SWIPT)-pairing, and [14] introduced a simplified automated strategy for optimal relay selection to enhance spectral and energy efficiency.

The deployment of multi-antenna technique in cooperative communication networks provides additional spatial diversity, thereby overcoming the capacity constraints characteristic of conventional cooperative architectures. For a multi-antenna two-way relay network employing NOMA, ref. [15] derived closed-form expressions for outage probability and diversity order and designed an power allocation scheme to minimize outage probability. Three antenna selection schemes were proposed in [16] for full-duplex cooperative NOMA networks, with results showing that the quality-of-service scheme achieves near optimal performance. Subsequently, an iterative algorithm optimizing the power splitting ratio and beamforming vectors was designed in [17] to maximize the energy efficiency of cooperative NOMA networks with energy harvesting capabilities. In [18], the authors proposed a cooperative NOMA network in multiple-input-multiple-output (MIMO) channels, developing a closed-form suboptimal algorithm to maximize the achievable rate for cell-edge user subject to power and rate constraints. The authors in [19] combined NOMA and multi-antenna technique in a full-duplex uplink cooperative network, employing zero-forcing at the base station to mitigate interference and deriving closed-form expression for outage probability.

### 1.2. Related Work

The above studies on multi-antenna cooperative NOMA networks assumed transmissions with infinite blocklength, enabling achievement of the theoretical channel capacity limit. However, in IoT applications requiring ultra-reliable low-latency communications (URLLC), short-packet transmissions are essential. In such scenarios, the finite blocklength effect becomes non-negligible and significantly impacts system performance [20,21]. The authors in [22] introduced a new metric, i.e., average block error rate (BLER), to evaluate short-packet communications. In a cooperative NOMA short-packet communications network, ref. [23] analyzed the average BLER performance for central and cell-edge users, demonstrating that cooperative relaying by the central user significantly enhances transmission reliability for the cell-edge user. When a full-duplex near user employing decode-and-forward (DF) protocol assists a far user, ref. [24] derived closed-form BLER expressions and demonstrated that short-packet transmission requires stricter power allocation than long-packet transmission to ensure reliable performance. In [25], channel coding ratios and power allocation coefficients were jointly optimized to maximize the throughput of cooperative NOMA networks. An adaptive hybrid relaying protocol for short-packet NOMA networks was proposed in [26], where a relay dynamically selects transmission modes to enhance weak-user performance. Closed-form and asymptotic BLER expressions were derived in [27] to characterize the performance of a novel partial decode-and-amplify NOMA scheme. The authors in [28] presented a low-complexity relay-sharing protocol that enables a single relay to simultaneously support two source destination pairs and significantly reduces encoding and decoding complexity. By adopting the transmit antenna selection scheme, ref. [29] demonstrated that increasing the number of antennas at the cellular transmitter significantly accelerates BLER convergence for cellular user. For NOMA networks with dynamic user pairing, the work in [30] focused on half-duplex relaying, whereas [31] studied full-duplex relaying.

However, the implementation of NOMA in practical scenarios is often challenged by hardware impairments, which can degrade system performance, particularly in short-packet communications [32,33]. For an uplink NOMA network with hardware impairments, ref. [34] conducted a comprehensive analysis of the average BLER and throughput. The combined of NOMA and reconfigurable intelligent surfaces (RIS) was examined in [35], revealing that hardware impairments have a lesser impact on users experiencing poorer channel conditions. For a cognitive NOMA network with hardware impairments, ref. [36] derived closed-form expressions for outage probability under the infinite blocklength transmission and average BLER under the finite blocklength transmission. The analytical and asymptotic expressions for average BLER were given in [37] to evaluate the reliability performance of RIS-assisted NOMA networks with hardware impairments. The authors in [38] analyzed the downlink performance of rate-splitting multiple access (RSMA) networks operating in the finite blocklength regime and derived closed-form expressions for the average BLER in the presence of practical impairments. Motivated by the above discussion, a clear research gap is identified in the performance analysis of multi-antenna cooperative NOMA networks that utilize short-packet communications in the presence of hardware impairments. Moreover, it is critical to investigate how key parameters, such as the number of transmit antennas and the blocklength, affect the system’s transmission reliability.

### 1.3. Contributions

Therefore, this work evaluates the performance of a multi-antenna cooperative NOMA network employing short-packet communications under hardware impairments. Unlike the single-antenna approach in [23], multi-antenna technique is employed to enhance spatial degrees of freedom for short-packet communications. In addition, practical hardware impairments are incorporated into the system model to enable a more realistic performance assessment. Compared with the relay-aided cooperative NOMA scheme presented in [29], the proposed system utilizes a near user to assist a multi-antenna source in cooperatively relaying signals to a far user. The main contributions of the paper are summarized as follows:

1. An analytical framework is proposed for modeling downlink multi-antenna cooperative NOMA networks with hardware impairments in a Nakagami-*m* fading environment, where short-packet transmission is adopted to lower communication delays. In particular, the direct link from the source to the near user and the relay link from the near user to the far user are combined by selective combining (SC) to enhance spectrum efficiency.
2. The maximum ratio transmission (MRT) scheme is employed to enhance the channel difference between the near and far users. Closed-form expressions for the average BLER and effective throughput are derived. Furthermore, the optimization of the blocklength is performed to maximize the effective throughput, subject to the constraints imposed on transmission latency and reliability.
3. Theoretical results are validated through extensive Monte Carlo simulations and show that the proposed scheme, utilizing a two-antenna source, achieves a significantly higher effective throughput. Specifically, at a transmit signal-to-noise ratio (SNR) of 33 dB, the proposed scheme attains a throughput improvement of up to 240% compared to the existing NOMA benchmark. Moreover, as the number of transmit antennas increases from 2 to 8, the performance advantage of the proposed scheme becomes particularly pronounced.

Table 1 summarizes the key distinctions between the contributions of this work and those of existing related studies.

### 1.4. Organization

The rest of this paper is summarized as follows. Section 2 details the system architecture of the multi-antenna cooperative NOMA network. Section 3 introduces newly derived analytical expressions for the average BLER and effective throughput, including the determination of an optimal blocklength that maximizes the effective throughput. Section 4 presents numerical results validating these analytical findings. Finally, Section 5 highlights the principal findings and contributions of this work. For notational convenience, a list of the fundamental parameters is provided in Table 2.

## 2. System Model

**Figure 1:**
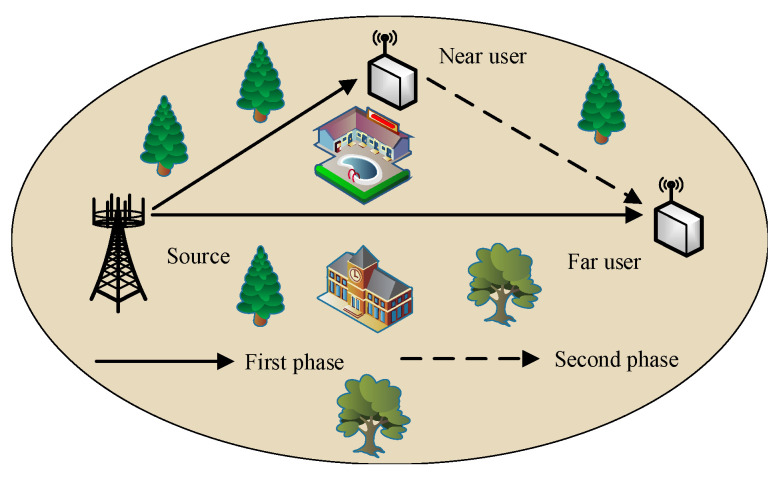
A multi-antenna cooperative NOMA network consists of a source, a near user, and a far user.

A multi-antenna cooperative NOMA network is considered, as illustrated in Figure 1. The network consists of a source (*S*) equipped with *N* antennas, a near user ($U_{1}$), and a far user ($U_{2}$), each employing a single antenna. This configuration is particularly relevant in practical applications, such as IoT scenarios, where multi-antenna access points communicate with two sensors, each constrained to a single antenna due to cost and size limitations [29]. To achieve low latency, the information transmitted from *S* to $U_{1}$ and $U_{2}$ is formatted into short-packet. As a result, the end-to-end delay is significantly shorter than the channel coherence time. Therefore, the channel coefficients are modeled as constant within each transmission block and independently varying across blocks [39]. Due to the half-duplex limitation of each node, each transmission block is divided into two phases. In the first phase, *S* broadcasts a superposed signal to $U_{1}$ and $U_{2}$ using the NOMA scheme. In the second phase, $U_{1}$ acts as a relay, forwarding the signal intended for $U_{2}$.

To exploit the benefits of multiple antennas at *S*, MRT is employed to improve the received SNR, thereby reducing bit error rates and enhancing data transmission efficiency. Furthermore, by precisely steering signals toward intended receivers, MRT can amplify the channel difference between the near and far users. Let $x_{s}=\sqrt{\alpha_{1}P}x_{1}+\sqrt{\alpha_{2}P}x_{2}$ be the superposed signal at *S*, where $\alpha_{1}$ and $\alpha_{2}$ are the transmit power allocation coefficients for $U_{1}$ and $U_{2}$, respectively, $x_{1}$ and $x_{2}$ are the signals intended for $U_{1}$ and $U_{2}$, respectively, and *P* is the transmit power of *S*. Since $U_{1}$ is the near user, the power allocation coefficients must satisfy $\alpha_{2}>\alpha_{1}$ and $\alpha_{1}+\alpha_{2}=1$. Then, the received signals at $U_{1}$ and $U_{2}$ during the first phase can be, respectively, expressed as

(1) $$y_{u_{1}}=h_{su_{1}}^{†}w\left(x_{s}+\tau_{ts}\right)+\tau_{ru_{1}}+n_{u_{1}},$$

and

(2) $$y_{u_{2}}^{1}=h_{su_{2}}^{†}w\left(x_{s}+\tau_{ts}\right)+\tau_{ru_{2}}^{1}+n_{u_{2}}^{1},$$

where $h_{su_{1}}$ is the N×1 channel vector for the $S-U_{1}$ link, and its entries follow independent and identically distributed (i.i.d.) Nakagami-*m* fading with mean $\Omega_{su_{1}}$ and fading parameter $m_{su_{1}}$, ${\left(\cdot \right)}^{†}$ is the conjugate transpose operator, w is the beamforming vector for MRT, $h_{su_{2}}$ is the N×1 channel vector for the $S-U_{2}$ link, and its entries follow i.i.d. Nakagami-*m* fading with mean $\Omega_{su_{2}}$ and fading parameter $m_{su_{2}}$; $\tau_{ts}∼CN\left(0,k_{1}^{2}P\right)$ is distortion noise caused by impairments at *S*; $\tau_{ru_{1}}∼CN\left(0,k_{2}^{2}P{\left|h_{su_{1}}^{†}w\right|}^{2}\right)$ and $\tau_{ru_{2}}^{1}∼CN\left(0,k_{2}^{2}P{\left|h_{su_{2}}^{†}w\right|}^{2}\right)$, respectively, are distortion noises caused by impairments at $U_{1}$ and $U_{2}$; $n_{u_{1}}$ and $n_{u_{2}}^{1}$, respectively, are the additive white Gaussian noise (AWGN) at $U_{1}$ and $U_{2}$ with variance $\sigma^{2}$. The parameters $k_{1}$ and $k_{2}$ characterize the levels of impairments at the transmitter and receiver hardware, respectively, which can be measured as error vector magnitudes (EVMs). w can be designed as $w=\frac{h_{su_{1}}}{\sqrt{h_{su_{1}}^{†}h_{su_{1}}}}$ for improving the reception performance of $U_{1}$, since $U_{2}$ depends on cooperation from $U_{1}$.

Following the NOMA principle, $U_{1}$ first decodes $x_{2}$ with the signal-to-noise-plus-interference ratio (SINR)

(3) $$\gamma_{1,2}=\frac{{\left|h_{su_{1}}^{†}w\right|}^{2}\alpha_{2}\lambda }{{\left|h_{su_{1}}^{†}w\right|}^{2}\lambda \left(\alpha_{1}+k_{1}^{2}+k_{2}^{2}\right)+1},$$

where $\lambda =\frac{P}{\sigma^{2}}$ is the transmit SNR. As given in [22], the instantaneous BLER for decoding $x_{2}$ at $U_{1}$ can be expressed as (The instantaneous BLER defines the relationship between reliability, blocklength, and the instantaneous received SNR in short-packet communications. Furthermore, its primary objective is to provide a rigorous mathematical foundation for deriving key performance metrics, such as the average BLER and effective throughput).

(4) $$\varepsilon_{1,2}\approx \Psi \left(\gamma_{1,2},b_{2},L\right),$$

where Ψγ1,2,b2,L=ΔQln2log21+γ1,2−b2b2LLVγ1,2Vγ1,2LL, $b_{2}$ is the number of information bits encoded in $x_{2}$, *L* is the blocklength, $V\left(\gamma \right)=1-{\left(1+\gamma \right)}^{-2}$, and $Q\left(x\right)=\frac{1}{2\pi }\int_{x}^{\infty }e^{-\frac{t^{2}}{2}}dt$ is the Gaussian *Q*-function. After decoding $x_{2}$, $U_{1}$ reconstructs it using the known modulation and coding scheme, and subtracts it from the received superposition signal. Then, $U_{1}$ decodes $x_{1}$ with SINR

(5) $$\gamma_{1,1}=\frac{{\left|h_{su_{1}}^{†}w\right|}^{2}\alpha_{1}\lambda }{{\left|h_{su_{1}}^{†}w\right|}^{2}\lambda \left(k_{1}^{2}+k_{2}^{2}\right)+1}.$$

Thus, the instantaneous BLER at $U_{1}$ is expressed as

(6) $$\begin{array}{c} \varepsilon_{u_{1}}=\varepsilon_{1,2}+\left(1-\varepsilon_{1,2}\right)\varepsilon_{1,1}, \end{array}$$

where $\varepsilon_{1,1}\approx \Psi \left(\gamma_{1,1},b_{1},L\right)$ and $b_{1}$ is the number of information bits encoded in $x_{1}$.

In the first phase, $U_{2}$ directly decode its own message with SINR

(7) $$\gamma_{2,2}^{1}=\frac{{\left|h_{su_{2}}^{†}w\right|}^{2}\alpha_{2}\lambda }{{\left|h_{su_{2}}^{†}w\right|}^{2}\lambda \left(\alpha_{1}+k_{1}^{2}+k_{2}^{2}\right)+1}.$$

During the second phase, $U_{1}$ cooperatively relays $x_{2}$ to $U_{2}$ with transmit power *P*. Thus, the received signal at $U_{2}$ can be expressed as

(8) $$y_{u_{2}}^{2}=\sqrt{P}h_{u_{1}u_{2}}\left(x_{2}+\tau_{tu_{1}}\right)+\tau_{ru_{2}}^{2}+n_{u_{2}}^{2},$$

where $h_{u_{1}u_{2}}$ is the Nakagami-*m* channel coefficient for the $U_{1}-U_{2}$ link with mean $\Omega_{u_{1}u_{2}}$ and fading parameter $m_{u_{1}u_{2}}$, $\tau_{tu_{1}}∼CN\left(0,k_{1}^{2}\right)$ is distortion noise caused by impairments at $U_{1}$, $\tau_{ru_{2}}^{2}∼CN\left(0,k_{2}^{2}P{\left|h_{u_{1}u_{2}}\right|}^{2}\right)$ is distortion noise caused by impairments at $U_{2}$, and $n_{u_{2}}^{2}$ is the AWGN with variance $\sigma^{2}$. Accordingly, the received SINR to decode $x_{2}$ at $U_{2}$ during the second phase is given by

(9) $$\gamma_{2,2}^{2}=\frac{{\left|h_{u_{1}u_{2}}\right|}^{2}\lambda }{{\left|h_{u_{1}u_{2}}\right|}^{2}\lambda \left(k_{1}^{2}+k_{2}^{2}\right)+1}.$$

To improve the quality and reliability of the received signals at $U_{2}$, the signals from the first and second phases are combined using SC scheme. Then, the SINR for decoding $x_{2}$ is expressed as

(10) $$\gamma_{2,2}=\max\left(\gamma_{2,2}^{1},\gamma_{2,2}^{2}\right).$$

Thus, the instantaneous BLER at $U_{2}$ is expressed as

(11) $$\begin{array}{c} \varepsilon_{u_{2}}=\varepsilon_{1,2}\Psi \left(\gamma_{2,2}^{1},b_{2},L\right)+\left(1-\varepsilon_{1,2}\right)\Psi \left(\gamma_{2,2},b_{2},L\right). \end{array}$$

## 3. Performance Analysis

This section presents a comprehensive evaluation of the reliability and effectiveness of the multi-antenna cooperative NOMA network. Closed-form expressions are derived for the average BLER and effective throughput. Furthermore, the transmission blocklength is optimized to maximize the effective throughput.

### 3.1. Average BLER

The closed-form expression of the average BLER at $U_{1}$ can be expressed as

(12) $${\bar{\varepsilon }}_{u_{1}}\approx E\left(\varepsilon_{1,1}\right)+E\left(\varepsilon_{1,2}\right),$$

where $E\left(\cdot \right)$ is the expectation over all channel realizations, and $E\left(\varepsilon_{1,1}\right)$ and $E\left(\varepsilon_{1,2}\right)$ are given by

(13) $$\begin{array}{cc} E\left(\varepsilon_{1,1}\right) & =\left(\frac{1}{2}-g_{1}h_{1}\sqrt{L}\right)\left(U\left(p_{1}-\frac{\alpha_{1}}{k_{1}^{2}+k_{2}^{2}}\right)-U\left(\frac{\alpha_{1}}{k_{1}^{2}+k_{2}^{2}}-p_{1}\right)F_{{\left|h_{su_{1}}^{†}w\right|}^{2}}\left(\varphi_{1}\left(p_{1}\right)\right)\right)\\ \; & \;\;\;\;+\left(\frac{1}{2}+g_{1}h_{1}\sqrt{L}\right)\left(U\left(q_{1}-\frac{\alpha_{1}}{k_{1}^{2}+k_{2}^{2}}\right)-U\left(\frac{\alpha_{1}}{k_{1}^{2}+k_{2}^{2}}-q_{1}\right)F_{{\left|h_{su_{1}}^{†}w\right|}^{2}}\left(\varphi_{1}\left(q_{1}\right)\right)\right)\\ \; & \;\;\;\;-\sum_{s=0}^{Nm_{su_{1}}}\left(\begin{array}{c} Nm_{su_{1}}\\ s \end{array}\right){\left(\frac{m_{su_{1}}}{\Omega_{su_{1}}}\right)}^{Nm_{su_{1}}}\frac{{\left(-1\right)}^{Nm_{su_{1}}-s}g_{1}\alpha_{1}\sqrt{L}e^{\mu_{1}}\mu_{1}^{-s}}{\Gamma \left(Nm_{su_{1}}\right)\lambda^{Nm_{su_{1}}}{\left(k_{1}^{2}+k_{2}^{2}\right)}^{Nm_{su_{1}}+1}}\\ \; & \;\;\;\;\times U\left(\frac{\alpha_{1}}{k_{1}^{2}+k_{2}^{2}}-p_{1}\right)\left(\Gamma \left(s,\mu_{1}u_{1}\left(p_{1}\right)\right)-U\left(\frac{\alpha_{1}}{k_{1}^{2}+k_{2}^{2}}-q_{1}\right)\Gamma \left(s,\mu_{1}u_{1}\left(q_{1}\right)\right)\right) \end{array}$$

(14) $$\begin{array}{cc} \; & E\left(\varepsilon_{1,2}\right)\\ \; & =\left(\frac{1}{2}-g_{2}h_{2}\sqrt{L}\right)\left(U\left(p_{2}-\frac{\alpha_{2}}{\alpha_{1}+k_{1}^{2}+k_{2}^{2}}\right)-U\left(\frac{\alpha_{2}}{\alpha_{1}+k_{1}^{2}+k_{2}^{2}}-p_{2}\right)F_{{\left|h_{su_{1}}^{†}w\right|}^{2}}\left(\varphi_{2}\left(p_{2}\right)\right)\right)\\ \; & \;\;\;\;+\left(\frac{1}{2}+g_{2}h_{2}\sqrt{L}\right)\left(U\left(q_{2}-\frac{\alpha_{2}}{\alpha_{1}+k_{1}^{2}+k_{2}^{2}}\right)-U\left(\frac{\alpha_{2}}{\alpha_{1}+k_{1}^{2}+k_{2}^{2}}-q_{2}\right)F_{{\left|h_{su_{1}}^{†}w\right|}^{2}}\left(\varphi_{2}\left(q_{2}\right)\right)\right)\\ \; & \;\;\;\;-\sum_{s=0}^{Nm_{su_{1}}}\left(\begin{array}{c} Nm_{su_{1}}\\ s \end{array}\right){\left(\frac{m_{su_{1}}}{\Omega_{su_{1}}}\right)}^{Nm_{su_{1}}}\frac{{\left(-1\right)}^{Nm_{su_{1}}-s}g_{2}\alpha_{2}\sqrt{L}e^{\mu_{2}}\mu_{2}^{-s}}{\Gamma \left(Nm_{su_{1}}\right)\lambda^{Nm_{su_{1}}}{\left(\alpha_{1}+k_{1}^{2}+k_{2}^{2}\right)}^{Nm_{su_{1}}+1}}\\ \; & \;\;\;\;\times U\left(\frac{\alpha_{2}}{\alpha_{1}+k_{1}^{2}+k_{2}^{2}}-p_{2}\right)\left(\Gamma \left(s,\mu_{2}u_{2}\left(p_{2}\right)\right)-U\left(\frac{\alpha_{2}}{\alpha_{1}+k_{1}^{2}+k_{2}^{2}}-q_{2}\right)\Gamma \left(s,\mu_{2}u_{2}\left(q_{2}\right)\right)\right) \end{array}$$

with g1=12π22b1b1LL−1, h1=2b1b1LL−1, $p_{1}=h_{1}-\frac{1}{2g_{1}\sqrt{L}}$, $q_{1}=h_{1}+\frac{1}{2g_{1}\sqrt{L}}$, $\mu_{1}=\frac{m_{su_{1}}}{\Omega_{su_{1}}\lambda \left(k_{1}^{2}+k_{2}^{2}\right)}$, $\varphi_{1}\left(x\right)=\frac{x}{\lambda \left(\alpha_{1}-x\left(k_{1}^{2}+k_{2}^{2}\right)\right)}$, $u_{1}\left(x\right)=\lambda \left(k_{1}^{2}+k_{2}^{2}\right)\varphi_{1}\left(x\right)+1$, g2=12π22b2b2LL−1, h2=2b2b2LL−1, $p_{2}=h_{2}-\frac{1}{2g_{2}\sqrt{L}}$, $q_{2}=h_{2}+\frac{1}{2g_{2}\sqrt{L}}$, $\mu_{2}=\frac{m_{su_{1}}}{\Omega_{su_{1}}\lambda \left(\alpha_{1}+k_{1}^{2}+k_{2}^{2}\right)}$, $\varphi_{2}\left(x\right)=\frac{x}{\lambda \left(\alpha_{2}-x\left(\alpha_{1}+k_{1}^{2}+k_{2}^{2}\right)\right)}$, $u_{2}\left(x\right)=\lambda \left(\alpha_{1}+k_{1}^{2}+k_{2}^{2}\right)\varphi_{2}\left(x\right)+1$, $\Gamma \left(\alpha ,x\right)=\int_{x}^{\infty }e^{-t}t^{\alpha -1}dt$, $U\left(x\right)=\left\{\begin{array}{cc} 1, & x\geq 0\\ 0, & x<0 \end{array}\right.$, and $F_{{\left|h_{su_{1}}^{†}w\right|}^{2}}\left(x\right)=1-\sum_{r=0}^{Nm_{su_{1}}}{\left(\frac{m_{su_{1}}x}{\Omega_{su_{1}}}\right)}^{r}\frac{1}{r!}e^{-\frac{m_{su_{1}}x}{\Omega_{su_{1}}}}$.

**Proof.**  The proof is given in Appendix A. □

The closed-form expression of the average BLER at $U_{2}$ can be expressed as

(15) $$\begin{array}{cc} {\bar{\varepsilon }}_{u_{2}} & =E\left(\varepsilon_{1,2}\right)E\left(\Psi \left(\gamma_{2,2}^{1},b_{2},L\right)\right)+\left(1-\right.E\left.\left(\varepsilon_{1,2}\right)\right)\\ \; & \;\;\;\;\times E\left(\Psi \left(\gamma_{2,2},b_{2},L\right)\right). \end{array}$$

By utilizing the probability density function (PDF) of ${\left|h_{su_{2}}^{†}w\right|}^{2}$, expressed as $f_{{\left|h_{su_{2}}^{†}w\right|}^{2}}\left(x\right)={\left(\frac{m_{su_{2}}}{\Omega_{su_{2}}}\right)}^{m_{su_{2}}}\frac{x^{m_{su_{2}}-1}}{\Gamma \left(m_{su_{2}}\right)}e^{-\frac{m_{su_{2}}x}{\Omega_{su_{2}}}}$ and following a procedure analogous to that detailed in Appendix A, $E\left(\Psi \left(\gamma_{2,2}^{1},b_{2},L\right)\right)$ is given by

(16) $$\begin{array}{cc} \; & E\left(\Psi \left(\gamma_{2,2}^{1},b_{2},L\right)\right)\\ \; & =\left(\frac{1}{2}-g_{2}h_{2}\sqrt{L}\right)\left(U\left(p_{2}-\frac{\alpha_{2}}{\alpha_{1}+k_{1}^{2}+k_{2}^{2}}\right)-U\left(\frac{\alpha_{2}}{\alpha_{1}+k_{1}^{2}+k_{2}^{2}}-p_{2}\right)F_{{\left|h_{su_{2}}^{†}w\right|}^{2}}\left(\varphi_{2}\left(p_{2}\right)\right)\right)\\ \; & +\left(\frac{1}{2}+g_{2}h_{2}\sqrt{L}\right)\left(U\left(q_{2}-\frac{\alpha_{2}}{\alpha_{1}+k_{1}^{2}+k_{2}^{2}}\right)-U\left(\frac{\alpha_{2}}{\alpha_{1}+k_{1}^{2}+k_{2}^{2}}-q_{2}\right)F_{{\left|h_{su_{2}}^{†}w\right|}^{2}}\left(\varphi_{2}\left(q_{2}\right)\right)\right)\\ \; & -\sum_{s=0}^{m_{su_{2}}}\left(\begin{array}{c} m_{su_{2}}\\ s \end{array}\right){\left(\frac{m_{su_{2}}}{\Omega_{su_{2}}}\right)}^{m_{su_{2}}}\frac{{\left(-1\right)}^{m_{su_{2}}-s}g_{2}\alpha_{2}\sqrt{L}e^{\mu_{3}}\mu_{3}^{-s}}{\Gamma \left(m_{su_{2}}\right)\lambda^{m_{su_{2}}}{\left(\alpha_{1}+k_{1}^{2}+k_{2}^{2}\right)}^{m_{su_{2}}+1}}U\left(\frac{\alpha_{2}}{\alpha_{1}+k_{1}^{2}+k_{2}^{2}}-p_{2}\right)\\ \; & \times \left(\Gamma \left(s,\mu_{3}u_{2}\left(p_{2}\right)\right)-U\left(\frac{\alpha_{2}}{\alpha_{1}+k_{1}^{2}+k_{2}^{2}}-q_{2}\right)\Gamma \left(s,\mu_{3}u_{2}\left(q_{2}\right)\right)\right) \end{array}$$

where $\mu_{3}=\frac{m_{su_{2}}}{\Omega_{su_{2}}\lambda \left(\alpha_{1}+k_{1}^{2}+k_{2}^{2}\right)}$, and $F_{{\left|h_{su_{2}}^{†}w\right|}^{2}}\left(x\right)=1-\sum_{r=0}^{m_{su_{2}}}{\left(\frac{m_{su_{2}}x}{\Omega_{su_{2}}}\right)}^{r}\frac{1}{r!}e^{-\frac{m_{su_{2}}x}{\Omega_{su_{2}}}}$. It is computationally challenging to derive the exact BLER of $U_{2}$ for the combined direct and relaying signals. Therefore, an approximation method from [23] is employed, i.e., $E\left(\Psi \left(\gamma_{2,2},b_{2},L\right)\right)\approx E\left(\Psi \left(\gamma_{2,2}^{1},b_{2},L\right)\right)E\left(\Psi \left(\gamma_{2,2}^{2},b_{2},L\right)\right)$. Then, similar to the derivation presented in Appendix A, $E\left(\Psi \left(\gamma_{2,2}^{2},b_{2},L\right)\right)$ is given by

(17) $$\begin{array}{cc} \; & E\left(\Psi \left(\gamma_{2,2}^{2},b_{2},L\right)\right)\\ \; & =\left(\frac{1}{2}-g_{2}h_{2}\sqrt{L}\right)\left(U\left(p_{2}-\frac{1}{k_{1}^{2}+k_{2}^{2}}\right)-U\left(\frac{1}{k_{1}^{2}+k_{2}^{2}}-p_{2}\right)F_{{\left|u_{1}u_{2}\right|}^{2}}\left(\varphi_{3}\left(p_{2}\right)\right)\right)\\ \; & \;\;\;\;+\left(\frac{1}{2}+g_{2}h_{2}\sqrt{L}\right)\left(U\left(q_{2}-\frac{1}{k_{1}^{2}+k_{2}^{2}}\right)-U\left(\frac{1}{k_{1}^{2}+k_{2}^{2}}-q_{2}\right)F_{{\left|u_{1}u_{2}\right|}^{2}}\left(\varphi_{3}\left(q_{2}\right)\right)\right)\\ \; & \;\;\;\;-\sum_{s=0}^{m_{u_{1}u_{2}}}\left(\begin{array}{c} m_{u_{1}u_{2}}\\ s \end{array}\right){\left(\frac{m_{u_{1}u_{2}}}{\Omega_{u_{1}u_{2}}}\right)}^{m_{u_{1}u_{2}}}\frac{{\left(-1\right)}^{m_{u_{1}u_{2}}-s}g_{2}\sqrt{L}e^{\mu_{4}}\mu_{4}^{-s}}{\Gamma \left(m_{u_{1}u_{2}}\right)\lambda^{m_{u_{1}u_{2}}}{\left(k_{1}^{2}+k_{2}^{2}\right)}^{m_{u_{1}u_{2}}+1}}\\ \; & \;\;\;\;\times U\left(\frac{1}{k_{1}^{2}+k_{2}^{2}}-p_{2}\right)\left(\Gamma \left(s,\mu_{4}u_{3}\left(p_{2}\right)\right)-U\left(\frac{1}{k_{1}^{2}+k_{2}^{2}}-q_{2}\right)\Gamma \left(s,\mu_{4}u_{3}\left(q_{2}\right)\right)\right) \end{array}$$

where $\varphi_{3}\left(x\right)=\frac{x}{\lambda \left(1-x\left(k_{1}^{2}+k_{2}^{2}\right)\right)}$, $u_{3}\left(x\right)=\lambda \left(k_{1}^{2}+k_{2}^{2}\right)\varphi_{3}\left(x\right)+1$, $\mu_{4}=\frac{m_{u_{1}u_{2}}}{\Omega_{u_{1}u_{2}}\lambda \left(k_{1}^{2}+k_{2}^{2}\right)}$, and $F_{{\left|u_{1}u_{2}\right|}^{2}}\left(x\right)=1-\sum_{r=0}^{m_{u_{1}u_{2}}}{\left(\frac{m_{u_{1}u_{2}}x}{\Omega_{u_{1}u_{2}}}\right)}^{r}\frac{1}{r!}e^{-\frac{m_{u_{1}u_{2}}x}{\Omega_{u_{1}u_{2}}}}$.

The closed-form expressions offer an efficient method for evaluating the impact of hardware impairments on the average BLER. In addition, note that $E\left(\varepsilon_{1,1}\right)$ is a decreasing function of $\gamma_{1,1}$. Consequently, increasing $\alpha_{1}$, which corresponds to allocating more power to $U_{1}$, reduces $E\left(\varepsilon_{1,1}\right)$. On the other hand, $E\left(\varepsilon_{1,2}\right)$ is a decreasing function of $\gamma_{1,2}$. Therefore, increasing $\alpha_{1}$ leads to an increase in $E\left(\varepsilon_{1,2}\right)$. This implies that allocating more power to $U_{1}$ does not always reduce its average BLER. However, the average BLER of $U_{2}$ increases as $\alpha_{1}$ increases. This is because $\gamma_{1,2}$ and $\gamma_{2,2}^{1}$ are decreasing functions of $\alpha_{1}$. Furthermore, hardware impairments impose an upper bound on the received SINRs at $U_{1}$ and $U_{2}$. This bound becomes more stringent as the levels of hardware impairments increases. As a result, the presence of hardware impairments reduces the network’s transmission reliability.

### 3.2. Effective Throughput

For short-packet communications, the network’s effective throughput depends on the transmission rate and the overall average BLER experienced by users. Therefore, the effective throughput of the multi-antenna cooperative NOMA network is expressed as

(18) $$T=\frac{b_{1}}{L}\left(1-{\bar{\varepsilon }}_{u_{1}}\right)+\frac{b_{2}}{L}\left(1-{\bar{\varepsilon }}_{u_{2}}\right).$$

For a fixed number of information bits, an increase in blocklength reduces the transmission rate, which in turn enhances reliability performance. However, a larger blocklength also decreases spectral efficiency, as more channel uses are required to transmit the same number of information bits. Therefore, there exists a trade-off in selecting the blocklength to maximize the effective throughput. An optimization problem is formulated to maximize the effective throughput with respect to the blocklength, subject to reliability and transmission latency constraints, as follows:

(19) $$\begin{array}{cc} \max_{L}\;\;\;\; & T,\\ s.t.\;\;\;\; & {\bar{\varepsilon }}_{u_{i}}\leq {\bar{\varepsilon }}_{\max},i=1,2,\\ \;\;\;\; & 1\leq L\leq L_{\max}\\ \;\;\;\; & L\in N^{+}, \end{array}$$

where ${\bar{\varepsilon }}_{\max}$ represents the required reliability, $L_{\max}$ represents the transmission latency requirement, and $N^{+}$ is the non-negative integer set. In short-packet communications where error rate is characteristically low, higher-order error terms can be effectively omitted from the average BLER [34,35]. Thus, ${\bar{\varepsilon }}_{u_{2}}$ can be approximated as $E\left(\varepsilon_{2,2}\right)$. It can be observed that as the blocklength increases, the instantaneous BLER monotonically decreases since $\frac{\partial \varepsilon_{u_{i}}}{\partial L}<0$. Consequently, the average BLER also exhibits a decreasing trend with increasing the blocklength. Therefore, the reliability requirement can be mathematically expressed by an inequality $L\geq \max\left(\left\lceil L_{1}\right\rceil ,\left\lceil L_{2}\right\rceil \right)$, where $\left\lceil \cdot \right\rceil$ is the ceiling operation, $L_{1}$ is the solution of ${\bar{\varepsilon }}_{u_{1}}={\bar{\varepsilon }}_{\max}$, and $L_{2}$ is the solution of ${\bar{\varepsilon }}_{u_{2}}={\bar{\varepsilon }}_{\max}$. However, closed-form expressions of $L_{1}$ and $L_{2}$ are unavailable due to the complexity of ${\bar{\varepsilon }}_{u_{1}}$ and ${\bar{\varepsilon }}_{u_{2}}$. Alternatively, $L_{1}$ and $L_{2}$ can be obtained through numerical methods, such as the golden-section search method. Thus, the optimal blocklength that maximizes effective throughput only exists under the condition $\max\left(\left\lceil L_{1}\right\rceil ,\left\lceil L_{2}\right\rceil \right)\leq L_{\max}$. When $\max\left(\left\lceil L_{1}\right\rceil ,\left\lceil L_{2}\right\rceil \right)\leq L_{\max}$, the effective throughput maximization problem can be further written as

(20) $$\begin{array}{cc} \max_{L}\;\;\;\; & T,\\ s.t.\;\;\;\; & \max\left(\left\lceil L_{1}\right\rceil ,\left\lceil L_{2}\right\rceil \right)\leq L\leq L_{\max},\\ \;\;\;\; & L\in N^{+}. \end{array}$$

The demonstration that *T* is a quasi-concave function of *L* begins by examining the second-order derivative of $\varepsilon_{i,j}$$\left(j=1,2\right)$ with respect to *L*, denoted as

(21) $$\frac{\partial^{2}\varepsilon_{i,j}}{\partial L^{2}}=\frac{\partial^{2}\varepsilon_{i,j}}{\partial \phi^{2}}{\left(\frac{\partial \phi }{\partial L}\right)}^{2}+\frac{\partial \varepsilon_{i,j}}{\partial \phi }\frac{\partial^{2}\phi }{\partial L^{2}},$$

where ϕ=ln2log21+γi,j−bjbjLLVγi,jVγi,jLL. Note that $\varepsilon_{i,j}$ is typically much smaller than 0.5 to ensure high reliability [40]. Consequently, $\phi =Q^{-1}\left(\varepsilon_{i,j}\right)>0$, where $Q^{-1}\left(x\right)$ is the inverse of the Gaussian *Q*-function. Moreover, the first and second derivatives of $\varepsilon_{i,j}$ with respect to ϕ satisfy $\frac{\partial \varepsilon_{i,j}}{\partial \phi }=-\frac{1}{\sqrt{2\pi }}e^{-\frac{\phi^{2}}{2}}<0$, and $\frac{\partial^{2}\varepsilon_{i,j}}{\partial \phi^{2}}=\frac{\phi }{\sqrt{2\pi }}e^{-\frac{\phi^{2}}{2}}>0$. The subsequent analysis focuses on the partial derivatives of ϕ with respect to *L*. Specifically, the first derivative $\frac{\partial \phi }{\partial L}$ and the second derivative $\frac{\partial^{2}\phi }{\partial L^{2}}$ are given by

(22) $$\frac{\partial \phi }{\partial L}=\ln2\frac{L\log_{2}\left(1+\gamma_{i,j}\right)+b_{j}}{2L^{\frac{3}{2}}\sqrt{V\left(\gamma_{i,j}\right)}},$$

(23) $$\frac{\partial^{2}\phi }{\partial L^{2}}=-\ln2\frac{L\log_{2}\left(1+\gamma_{i,j}\right)+3b_{j}}{4L^{\frac{5}{2}}\sqrt{V\left(\gamma_{i,j}\right)}}.$$

It is clear from (22) and (23) that $\frac{\partial \phi }{\partial L}>0$ and $\frac{\partial^{2}\phi }{\partial L^{2}}<0$. Thus, $\varepsilon_{i,j}$ is a convex decreasing function with respect to *L*. Following the Leibniz integral rule, ${\bar{\varepsilon }}_{u_{i}}$ maintains convex characteristics when *L* varies. As a result, the objective function *T* is a quasi-concave function of *L*.

Based on the above discussion, the optimal blocklength for effective throughput maximization can be expressed as

(24) $$\begin{array}{c} L^{*}=\arg\max_{L\in \left\{\left[\left\lceil L^{\#}\right\rceil ,\left\lfloor L^{\#}\right\rfloor \right]\cap \left[\max\left(\left\lceil L_{1}\right\rceil ,\left\lceil L_{2}\right\rceil \right),L_{\max}\right]\max\left(\left\lceil L_{1}\right\rceil ,\left\lceil L_{2}\right\rceil \right),L_{\max}\right\}}T, \end{array}$$

where $L^{\#}$ is the solution of $\frac{\partial T}{\partial L}=0$, and $\left\lfloor \cdot \right\rfloor$ is the floor operation.

## 4. Simulation Results

In this section, representative numerical results obtained from Matlab are presented to demonstrate the efficiency of the proposed scheme compared to the benchmark scheme. Additionally, these results reveal key insights regarding average BLER and effective throughput. Unless otherwise stated, the simulation parameters are given in Table 3.

**Figure 2:**
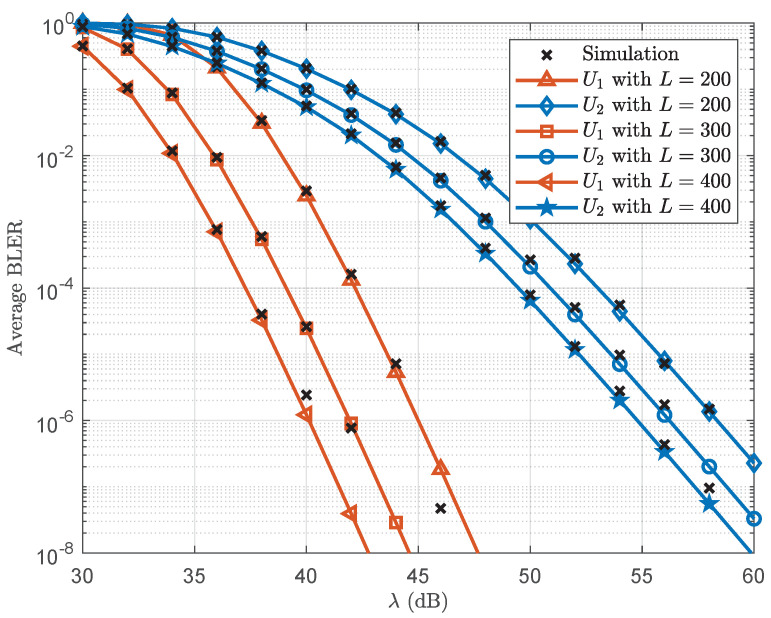
Average BLER versus λ.

Figure 2 plots the average BLER versus λ. First, the simulation results are observed to align precisely with the analytical curves, confirming the validity of derived analysis. Moreover, as expected, increasing the transmit SNR reduces the average BLER for $U_{1}$ and $U_{2}$. In addition, extending the blocklength enhances reliability performance. This improvement occurs because, for a fixed amount of transmitted information, a longer blocklength lowers the transmission rate.

**Figure 3:**
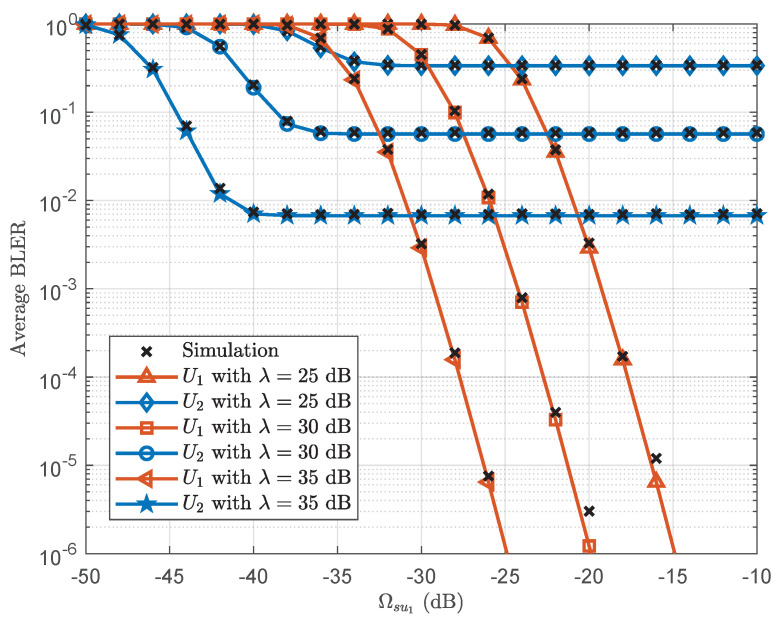
Average BLER versus $\Omega_{su_{1}}$ with L=400.

Figure 3 plots the average BLER versus $\Omega_{su_{1}}$ with L=400. It can be observed that the average BLER of $U_{1}$ decreases as $\Omega_{su_{1}}$ increases. However, the average BLER of $U_{2}$ first decreases and eventually approaches to a floor as $\Omega_{su_{1}}$ increases. This is because when $\Omega_{su_{1}}$ is small, increasing $\Omega_{su_{1}}$ reduces the average BLER of $U_{1}$ for the decoded signal $x_{2}$, which consequently decreases the average BLER of $U_{2}$. However, when $\Omega_{su_{1}}$ becomes sufficiently large, the average BLER of $U_{2}$ for the decoded signal $x_{2}$ becomes a performance bottleneck.

**Figure 4:**
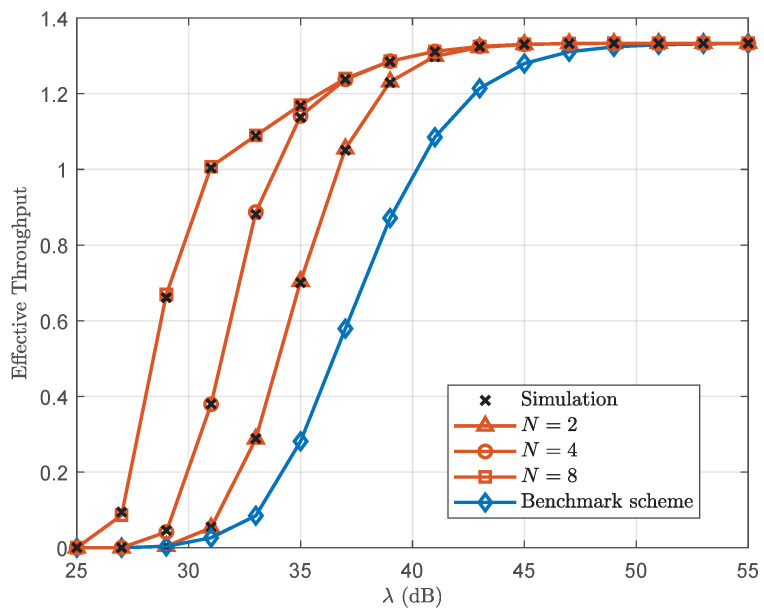
Effective throughput versus λ with L=300.

Figure 4 plots the effective throughput versus λ with L=300. First, it can be seen that as the transmit SNR increases, the effective throughput increases and eventually saturates to a constant value. This saturation occurs because, at high SNR region, the effective throughput becomes constrained by the transmission rate rather than channel conditions. Moreover, the proposed scheme demonstrates substantial performance gains over the benchmark scheme in [23], where the source is equipped with a single antenna. Specifically, the proposed scheme, with a two-antenna source configuration, achieves a superior effective throughput, reaching up to 240% at a transmit SNR of 33 dB, compared to the benchmark scheme. As the number of transmit antennas increases from 2 to 8, the performance advantage of the proposed scheme becomes particularly pronounced.

**Figure 5:**
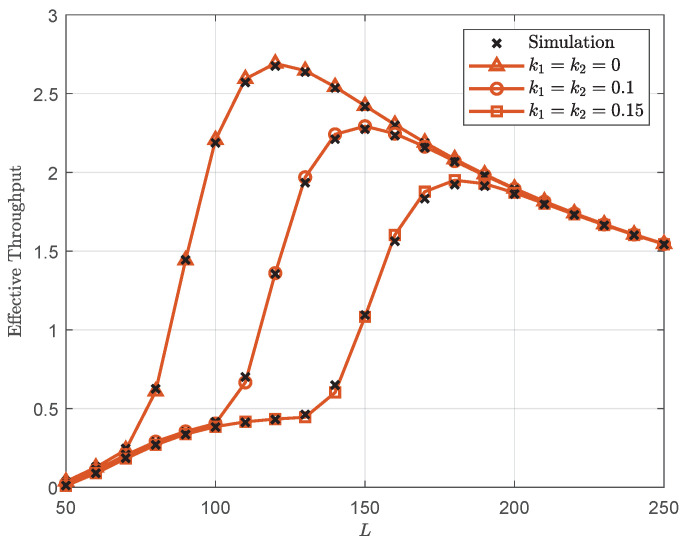
Effective throughput versus *L* with λ=40 dB.

Figure 5 plots the effective throughput versus *L* with λ=40 dB. It can be seen that the effective throughput initially increases with increasing *L* up to an optimal value, beyond which it begins to decrease. This behavior occurs because for *L* values below the optimal value, the effective throughput is limited by a higher average BLER. In contrast, for *L* values above the optimal value, the effective throughput reduction stems from lower transmission rates. Consequently, a tradeoff between decoding error and transmission latency must be considered by the designer. Furthermore, the optimal blocklength decreases with improved hardware quality (i.e., diminishing hardware impairments). This relationship arises because reduced hardware impairments lower the probability of decoding errors, which consequently permits the use of shorter blocklengths to minimize transmission latency without compromising reliability.

**Figure 6:**
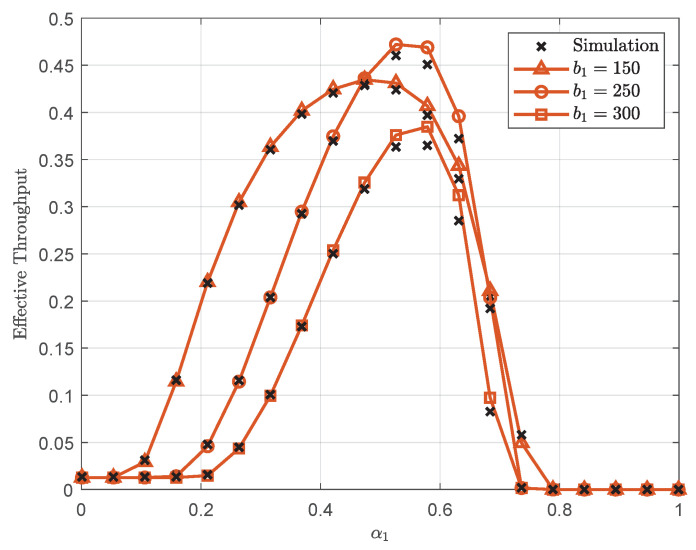
Effective throughput versus $\alpha_{1}$ with λ=30 dB, L=300, and N=2.

Figure 6 plots the effective throughput versus $\alpha_{1}$ with λ=30 dB, L=300, and N=2. It can be seen that the effective throughput first increases to a maximum at an optimal $\alpha_{1}$ before decreasing as $\alpha_{1}$ increases further. This behavior results from two competing error rate effects at $U_{1}$. When $\alpha_{1}$ is too small, the higher decoding error rate for signal $x_{1}$ limits the effective throughput. When $\alpha_{1}$ is too large, the increased decoding error rate for signal $x_{2}$ becomes the limiting factor. Consequently, the performance of $U_{1}$ is critically dependent on $\alpha_{1}$, which should be carefully designed to balance self-signal demodulation and SIC. Furthermore, the optimal $\alpha_{1}$ decreases as $b_{1}$ decreases. This phenomenon occurs because decreasing $b_{1}$ reduces the achievable rate for signal $x_{1}$, consequently requiring less power allocation to $U_{1}$ to maintain reliable transmission.

## 5. Conclusions

In this work, the performance of short-packet communications was examined in multi-antenna cooperative NOMA networks, where the multi-antenna source transmits superposition-coded NOMA signals to a pair of users. In particular, the near user operates as a DF relay, utilizing SIC to recover and then relay the far user’s intended message. Furthermore, MRT is employed at the source and SC is adopted at the far user. Under the effect of hardware impairments, closed-form expressions for the average BLER and effective throughput were derived. To maximize the effective throughput, the optimal blocklength was determined. Extensive Monte Carlo simulations validated the theoretical analysis, demonstrating that the proposed NOMA scheme achieves a higher effective throughput than the existing NOMA scheme when implemented with multiple antennas at the source. This work is particularly suited for mission-critical applications such as remote surgery, autonomous vehicles, and emergency response systems, where short-packet transmission combined with cooperative NOMA ensures minimal latency and high reliability. Future work can explore the integration of RIS with multi-antenna cooperative NOMA to further enhance the coverage and reliability of short-packet communications.

## Figures and Tables

**Table 1:** Comparison of this work with related papers.

	[13,14]	[23,24]	[26]	[30,31]	[34]	[35]	This Work
NOMA	✓	✓	✓	✓	✓	✓	✓
Cooperative transmission	✓	✓	✓	✓			✓
Short-packet communications		✓	✓	✓	✓	✓	✓
Hardware impairments					✓	✓	✓
Average BLER		✓		✓	✓	✓	✓
Effective throughput maximization					✓		✓

**Table 2:** List of fundamental parameters.

Symbol	Description
*N*	The number of antennas at *S*
*L*	The blocklength for short-packet communications
$b_{1}$	The number of information bits for $U_{1}$
$b_{2}$	The number of information bits for $U_{2}$
*P*	The transmit power at transmitters
$\sigma^{2}$	The variance of AWGN at receivers
$k_{1}$	The level of hardware impairments at transmitters
$k_{2}$	The level of hardware impairments at receivers
$\alpha_{1}$	The power allocation factor for $U_{1}$
$\alpha_{2}$	The power allocation factor for $U_{2}$
$h_{su_{1}}$	The channel vector from *S* to $U_{1}$
$h_{su_{2}}$	The channel vector from *S* to $U_{2}$
$h_{u_{1}u_{2}}$	The channel coefficient from $U_{1}$ to $U_{2}$
w	The beamforming vector for MRT
$\Omega_{su_{1}}$	The fading mean of each entity in $h_{su_{1}}$
$m_{su_{1}}$	The fading parameter of each entity in $h_{su_{1}}$
$\Omega_{su_{2}}$	The fading mean of each entity in $h_{su_{2}}$
$m_{su_{2}}$	The fading parameter of each entity in $h_{su_{2}}$
$\Omega_{u_{1}u_{2}}$	The fading mean of $h_{u_{1}u_{2}}$
$m_{u_{1}u_{2}}$	The fading parameter of $h_{u_{1}u_{2}}$
$\varepsilon_{u_{1}}$	The instantaneous BLER at $U_{1}$
$\varepsilon_{u_{2}}$	The instantaneous BLER at $U_{2}$
${\bar{\varepsilon }}_{u_{1}}$	The average BLER at $U_{1}$
${\bar{\varepsilon }}_{u_{2}}$	The average BLER at $U_{2}$
*T*	The effective throughput

**Table 3:** List of simulation parameters.

Parameter	Value
Number of antennas	N=4
Levels of hardware impairments	$k_{1}=k_{2}=0.1$
Fading parameters	$m_{su_{1}}=m_{su_{2}}=m_{u_{1}u_{2}}=2$
Fading means	$\Omega_{su_{1}}=-30$ dB, $\Omega_{su_{2}}=-50$ dB, $\Omega_{u_{1}u_{2}}=-40$ dB
Number of information bits	$b_{1}=300$, $b_{2}=100$
Power allocation coefficient	$\alpha_{1}=0.2$

## Data Availability

The raw data supporting the conclusions of this article will be made available by the corresponding author on request.

## Appendix A

According to (6), the average BLER at $U_{1}$ can be expressed as

(A1) $$\begin{array}{cc} {\bar{\varepsilon }}_{u_{1}} & =E\left(\varepsilon_{1,2}+\left(1-\varepsilon_{1,2}\right)\varepsilon_{1,1}\right)\\ \; & \overset{\left(a\right)}{\approx }E\left(\varepsilon_{1,2}\right)+E\left(\varepsilon_{1,1}\right), \end{array}$$

where step $\left(a\right)$ is due to the fact that the error rate is generally very low, for instance, $10^{-5}$ in short-packet communications scenarios [34]. According to (5), $E\left(\varepsilon_{1,1}\right)$ can be derived as

(A2) $$\begin{array}{cc} \; & E\left(\varepsilon_{1,1}\right)=\int_{0}^{\infty }\vartheta \left(\frac{\alpha_{1}\lambda x}{\lambda \left(k_{1}^{2}+k_{2}^{2}\right)x+1}\right)f_{{\left|h_{su_{1}}^{†}w\right|}^{2}}\left(x\right)dx, \end{array}$$

where $\vartheta \left(\gamma \right)=\left\{\begin{array}{cc} 1, & \gamma \leq p_{1},\\ \frac{1}{2}-g_{1}\sqrt{L}\left(\gamma -h_{1}\right), & p_{1}<\gamma <q_{1},\\ 0, & \gamma \geq q_{1}, \end{array}\right.$ is the approximation of the instantaneous BLER and $f_{{\left|h_{su_{1}}^{†}w\right|}^{2}}\left(x\right)={\left(\frac{m_{su_{1}}}{\Omega_{su_{1}}}\right)}^{Nm_{su_{1}}}\frac{x^{Nm_{su_{1}}-1}}{\Gamma \left(Nm_{su_{1}}\right)}e^{-\frac{m_{su_{1}}x}{\Omega_{su_{1}}}}$. To derive the closed-form expression for $E\left(\varepsilon_{1,1}\right)$, three distinct cases are considered.

Case 1: When $\frac{\alpha_{1}}{k_{1}^{2}+k_{2}^{2}}\leq p_{1}$, $\vartheta \left(\frac{\alpha_{1}\lambda x}{\lambda \left(k_{1}^{2}+k_{2}^{2}\right)x+1}\right)=1$ and $E\left(\varepsilon_{1,1}\right)=1$.

Case 2: When $\frac{\alpha_{1}}{k_{1}^{2}+k_{2}^{2}}\geq q_{1}$, $\vartheta \left(x\right)$ can be expressed as

(A3) $$\begin{array}{c} \vartheta \left(x\right)=\left\{\begin{array}{cc} 1, & x\leq \varphi_{1}\left(p_{1}\right),\\ \frac{1}{2}-g_{1}\sqrt{L}\left(g\left(x\right)-h_{1}\right), & \varphi_{1}\left(p_{1}\right)<x<\varphi_{1}\left(q_{1}\right),\\ 0, & x\geq \varphi_{1}\left(q_{1}\right), \end{array}\right. \end{array}$$

where $g\left(x\right)=\frac{\alpha_{1}\lambda x}{\lambda \left(k_{1}^{2}+k_{2}^{2}\right)x+1}$.

Substituting (A3) into (A2), $E\left(\varepsilon_{1,1}\right)$ can be expressed as

(A4) $$\begin{array}{cc} E\left(\varepsilon_{1,1}\right) & =\left(\frac{1}{2}+g_{1}h_{1}\sqrt{L}\right)F_{{\left|h_{su_{1}}^{†}w\right|}^{2}}\left(\varphi_{1}\left(q_{1}\right)\right)+\left(\frac{1}{2}-g_{1}h_{1}\sqrt{L}\right)F_{{\left|h_{su_{1}}^{†}w\right|}^{2}}\left(\varphi_{1}\left(p_{1}\right)\right)\\ \; & \;\;\;\;-\underset{\Phi_{1}}{\underset{︸}{\int_{\varphi_{1}\left(p_{1}\right)}^{\varphi_{1}\left(q_{1}\right)}\frac{g_{1}\alpha_{1}\lambda \sqrt{L}x}{\lambda \left(k_{1}^{2}+k_{2}^{2}\right)x+1}{\left(\frac{m_{su_{1}}}{\Omega_{su_{1}}}\right)}^{Nm_{su_{1}}}\frac{x^{Nm_{su_{1}}-1}}{\Gamma \left(Nm_{su_{1}}\right)}e^{-\frac{m_{su_{1}}x}{\Omega_{su_{1}}}}dx}} \end{array}$$

Utilizing [41], p. 3.381.3, $\Phi_{1}$ is derived as

(A5) $$\begin{array}{cc} \Phi_{1} & =\sum_{s=0}^{Nm_{su_{1}}}\left(\begin{array}{c} \;\;Nm_{su_{1}}\;\;\\ \;\;s\;\; \end{array}\right)\frac{{\left(-1\right)}^{Nm_{su_{1}}-s}\mu_{1}^{-s}g_{1}\alpha_{1}\sqrt{L}e^{\mu_{1}}}{\Gamma \left(Nm_{su_{1}}\right)\lambda^{Nm_{su_{1}}}{\left(k_{1}^{2}+k_{2}^{2}\right)}^{Nm_{su_{1}}+1}}\\ \; & \;\;\;\;\times {\left(\frac{m_{su_{1}}}{\Omega_{su_{1}}}\right)}^{Nm_{su_{1}}}\left(\Gamma \left(s,\mu_{1}u_{1}\left(p_{1}\right)\right)-\Gamma \left(s,\mu_{1}u_{1}\left(q_{1}\right)\right)\right). \end{array}$$

Case 3: When $p_{1}<\frac{\alpha_{1}}{k_{1}^{2}+k_{2}^{2}}<q_{1}$, $\vartheta \left(x\right)$ can be expressed as

(A6) $$\vartheta \left(x\right)=\left\{\begin{array}{cc} 1, & x\leq \varphi \left(p_{1}\right),\\ \frac{1}{2}-g_{1}\sqrt{L}\left(g\left(x\right)-h_{1}\right), & \varphi \left(p_{1}\right)<x, \end{array}\right.$$

Substituting (A6) into (A2), $E\left(\varepsilon_{1,1}\right)$ can be expressed as

(A7) $$\begin{array}{cc} E\left(\varepsilon_{1,1}\right) & =\int_{0}^{\varphi_{1}\left(p_{1}\right)}f_{{\left|h_{su_{1}}^{†}w\right|}^{2}}\left(x\right)dx+\int_{\varphi_{1}\left(p_{1}\right)}^{\infty }\vartheta \left(x\right)f_{{\left|h_{su_{1}}^{†}w\right|}^{2}}\left(x\right)dx. \end{array}$$

By applying an analytical approach analogous to that employed in Case 2, the closed-form expression for (A7) is derived as

(A8) $$\begin{array}{cc} E\left(\varepsilon_{1,1}\right) & =\frac{1}{2}+g_{1}h_{1}\sqrt{L}+\left(\frac{1}{2}-g_{1}h_{1}\sqrt{L}\right)F_{{\left|h_{su_{1}}^{†}w\right|}^{2}}\left(\varphi_{1}\left(p_{1}\right)\right)\\ \; & \;\;\;\;-\sum_{s=0}^{Nm_{su_{1}}}\left(\begin{array}{c} Nm_{su_{1}}\\ s \end{array}\right)\frac{{\left(-1\right)}^{Nm_{su_{1}}-s}g_{1}\alpha_{1}\sqrt{L}e^{\mu_{1}}}{\Gamma \left(Nm_{su_{1}}\right){\left(k_{1}^{2}+k_{2}^{2}\right)}^{Nm_{su_{1}}+1}}\\ \; & \;\;\;\;\times \frac{\mu_{1}^{-s}}{\lambda^{Nm_{su_{1}}}}{\left(\frac{m_{su_{1}}}{\Omega_{su_{1}}}\right)}^{Nm_{su_{1}}}\Gamma \left(s,\mu_{1}u_{1}\left(p_{1}\right)\right). \end{array}$$

By integrating the three cases discussed above, the closed-form expression for $E\left(\varepsilon_{1,1}\right)$ is derived in (13). Subsequently, $E\left(\varepsilon_{1,2}\right)$ can be directly derived using a similar analytical approach as applied to $E\left(\varepsilon_{1,1}\right)$, as shown in (14).
